# Incidents of Animal Life

**Published:** 1887-03-05

**Authors:** F. O. Morris

**Affiliations:** Rector of Nunburnholme, Yorkshire; author of "British Birds," etc.


### INCIDENTS OF ANIMAL LIFE.

COLLATED BY THE REV. F. O. MORRIS, B.A.,
Rector of Nunburnholme, Yorkshire; author of "British Birds," etc.

"He prayeth best, who loveth best
All things both great and small;
For the dear God who loveth us,
He made and loveth all."—COLERIDGE.

A VERY touching incident has just been related to me by an eye-witness. A small white dog, quite a young one, was frisking and barking, and jumping up at a horse in a carrier's cart going down Friar Street, Reading, when suddenly the dog stumbled, and the horse involuntarily stepped on it, breaking both its hind legs. The horse, evidently conscious of what he had done, immediately stood still, and, bending down his head, began licking the little sufferer. The man picked it up and carried it into the yard of a surgeon close by. Mr. F. pronounced it a hopeless case, and that it must be shot. While he was examining its leg, the faithful animal was licking his kind master's hands. The big tears rolled down his cheeks as he said, "What will the horses do without it? they are such friends, and I have the mother of the puppy at home." The dear horse was meanwhile looking wistfully up the yard towards its little favourite, uttering mournful sounds. Another proof of the strong affection and intelligence of animals. What a lesson to the unfeeling and the cruel!

